# Association between lifestyle, gender and risk for developing end-stage renal failure in IgA nephropathy: a case-control study within 10 years

**DOI:** 10.1080/0886022X.2019.1635029

**Published:** 2019-10-03

**Authors:** Pei Pei Huang, Dan Hua Shu, Zhen Su, Sheng Nan Luo, Fei Fei Xu, Fan Lin

**Affiliations:** a Department of Nephrology, the First Affiliated Hospital of Wenzhou Medical University, Wenzhou, China;; b Department of Obstetrics, the First Affiliated Hospital of Wenzhou Medical University, Wenzhou, China

**Keywords:** Alcohol drinking, exercise, smoking, IgA, renal failure

## Abstract

**Purpose:** To investigate the potential association between lifestyles, including cigarette smoking, alcohol consumption, and physical exercise at the time of biopsy and the risk for developing end-stage renal failure (ESRF) among IgA nephropathy (IgAN) patients within 10 years.

**Methods:** A case–control study was carried out. Seventy-seven ESRF patients with the primary cause of IgAN were enrolled as cases. Seventy-seven IgAN patients who had not progressed to ESRF after being diagnosed for over 10 years served as controls. Smoking, alcohol consumption and physical exercise related data and baseline clinical features were collected from their medical records and confirmed by phone calls.

**Results:** The case group had higher proportions of males, smokers, drinkers, and physical inactivity individuals than the controls had. Alcohol drinking history (/1 year, OR 1.32, *p* < .05) is independently associated with an increased risk of ESRF, while physical exercise habits (OR 0.06, *p* < .05) associated with a decreased risk of ESRF in multivariate logistic analysis. Male gender, lower eGFR, and higher urinary protein at the time of biopsy were also independent risk factors. Moreover, male-non-exercise population seems to be more likely to progress to ESRF than others (male-exercise, female-exercise, and female-none-exercise populations).

**Conclusion:** Physical exercise should be encouraged in IgAN patients, especially in males, for a better renal outcome. Alcohol cessation might have a renal survival benefit in IgAN patients.

## Introduction

Immunoglobulin A nephropathy (IgAN) is one of the most common primary glomerular nephritis worldwide and is particularly prevalent in Asia. The proportion of IgAN progressing to end stage renal failure (ESRF) ranged from 10% to 25% within 10 years and 25% to 50% within 20 years [1]. Clinical characteristics, such as proteinuria and estimated glomerular filtration rate (eGFR) at the biopsy, have been reported relevant to the prognosis of IgAN [2]. On the other hand, unhealthy lifestyles like cigarette smoking and alcohol drinking, have recently drawn researchers’ attention with their adverse effects on the renal outcomes of chronic kidney diseases [3–5]. However, few Chinese population-based studies were available, which investigated the influences of daily lifestyles on IgAN renal outcomes. Therefore, the aim of this study is to investigate whether daily lifestyles, including smoking, alcohol drinking, and regular exercise, have impacts on the progression of IgAN to ESRF among the Chinese population, and provide a better lifestyle guide for IgAN patients.

## Methods

### Subject

**Figure 1.**
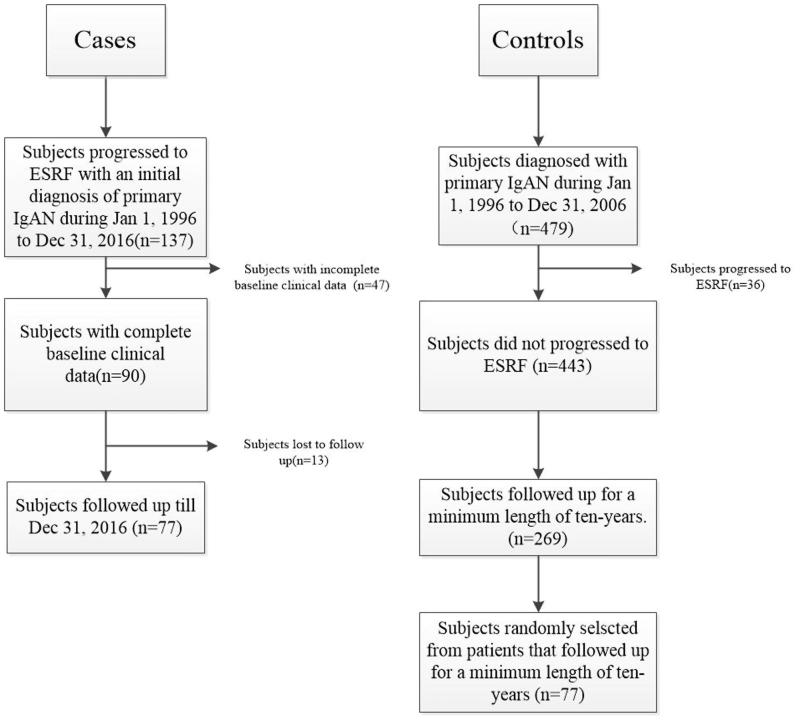
Flow diagram of subject assignment.

This was a retrospective case–control study. Cases were patients in the First Affiliated Hospital of Wenzhou Medical University, who had progressed to ESRF within ten years with an initial diagnosis of primary IgAN (*n* = 77), during 1 January 1996 to 31 December 2016. Those without baseline clinical data (*n* = 47) or cannot be contacted (*n* = 13) were excluded. There were no significant differences in the demographic between study participants and those who were excluded. Seventy-seven patients with a minimum of a 10-year IgAN history were selected randomly from outpatient as the control group, with the criterion of having not met the standard of ESRF. The subject assignment is shown in Figure 1. The diagnosis of IgAN was made by detection of mesangial deposits staining predominantly for IgA in immunofluorescence studies in patients without evidences of systemic lupus erythematosus, henoch-Schönlein purpura, chronic liver diseases, and rheumatoid arthritis. ESRF was defined as an irreversible decline of eGFR to under 15 mL·min^−1^· (1.73 m^2^)^−1^ or the start of renal replacement therapy.

### Data collection

Patients’ clinical data at the time of kidney biopsy and the subsequent therapeutic prescription were collected retrospectively from their medical records. Clinical data included age, gender, serum creatinine (Scr), 24-h urinary protein excretion or urinary protein-to-creatinine ratio, history of hypertension, smoking, and drinking status. The therapy included the use of prednisone, immunosuppression (except for prednisone) and RAS blockade. Estimated glomerular filtration rate (eGFR) was calculated by modified MDRD equation: eGFR [ml·min^−1^· (1.73 m^2^)^−1^] = 175 × [Scr, mg/dl]^−1.234^× [age]^−0.179^ × 0.79 [if female].

Smoking/drinking status of each patient at the time of biopsy including the amount of cigarette/alcohol consumed and starting/stopping dates were collected from medical records and reconfirmed by phone calls. The data of cigarette smoking was summed up as following variables: (1) cigarette smoking history (all patients): never smoked, ex-smoker, or current smoker; (2) number of cigarettes smoked per day; (3) cumulative quantity of cigarettes [packs × year] = pack (20 cigarettes)/day × duration of cigarette smoking (year). The data of alcohol consumption was studied for the following variables: (1) drinking history (all patients): never drunk, ex-drinker, or current drinker; (2) daily alcohol consumption; (3) drinking years. Alcohol consumption was calculated by the rough ethyl alcohol content of different kinds of wine: 100 mL white wine, 100 mL grape wine, and 100 mL beer contained 50 g, 8 g, and 3 g alcohol, respectively. Because we were most interested in whether a history of smoking/drinking was a risk factor of IgAN progression, current smokers/drinkers and ex-smokers/drinkers were enrolled into the same group (current/ex-smokers or current/ex-drinkers).

Detailed information of physical exercise prior to the biopsy was also obtained by questionnaire on phone calls. Exercise habits were defined as whether taking exercise regularly or not, including moderate activities (walking a mile without stopping, garden or yard work, and other activities) for at least three times per week, and vigorous activity (jogging, running or riding a bicycle or exercise bike, swimming, aerobics, dancing, calisthenics, and other activities) for one time per week. This study was performed with the written informed consent of all patients. The procedure was approved by the Ethics Committee of the First Affiliated Hospital of Wenzhou Medical University and been performed in accordance with the ethical standards laid down in the 2013 version of the Declaration of Helsinki.

### Statistical analysis

Descriptive analysis was done for all study variables. Continuous variables were expressed as mean (± standard deviation) for normally distributed data, or median (range) for abnormally distributed data. Categorical variables were described using frequencies (percentages). Differences across groups were assessed using Student’s *t* test, the Mann–Whitney *U* test, or the Chi-square test according to the sample. Spearman’s correlation coefficient was used to identify the relationship between living habits and baseline clinical data in cases and controls, respectively. Univariate logistic regression model was used to calculate the crude odds ratios (ORs) and 95% confidence intervals (CIs) of every single variable to the risk of ESRF. Adjusted ORs and 95% CIs of lifestyle variables were calculated using multivariate analysis, respectively, adjusted for confounding factors (including age, gender, eGFR, urinary protein at baseline, history of hypertension, and treatment of prednisone, and immunosuppression). We also used multivariate analysis in a model only contained confounding factors, which we called model 0, to investigate the role of these factors played in the progression of IgAN. Since variables of smoking and drinking status were strongly associated, they were further discussed together. But this was only analyzed in males due to the low number of females with the history of smoking or alcohol drinking (0 and 3patients, respectively). The ORs of a combined variable, gender-smoking and drinking, adjusted for age, eGFR, and proteinuria, were calculated by multivariate analysis. The adjusted ORs of gender-exercise combined variables were calculated using females (Model 1) or males (Model 2) without physical exercise habit as the reference category, and were also adjusted for age, eGFR, and proteinuria at biopsy. All statistical analyses were performed using SPSS version 22.0 for Windows (SPSS, Chicago, IL).

## Results

The case group was observed for 122.6 ± 47.3 months and the average renal survival time was 65.1 ± 43.8 months, while the control group was followed for 133.3 ± 15.3 months. Baseline data of each group are displayed in Table 1, and cases showed more severe clinical features at the time of biopsy. There were significant associations between gender and variables of cigarette smoking and alcohol consumption status in both groups, but no significant relationship was found between exercise habit and other variables (Table 2).

**Table 1.** Characteristics at biopsy of cases and controls.

	Cases (*n* = 77)	Controls (n = 77)		Cases (n = 77)	Controls (n = 77)
Sex (male/female)	55/22*	36/41	Cigarette smoking		
Age (years)	36.7 ± 10.4	36.1 ± 10.2	Current/ex-smokers [*n* (%)]	25 (32.5)*	11 (14.3)
Serum creatinine (μmol/l)	150.0 ± 68.4**	88.1 ± 27.5	Smoking length (years)^b^	5 (1–35)	2 (1–38)
eGFR	55.9 ± 25.6**	90.9 ± 29.8	Daily consumption (cigarette/day)^b^	10 (3–60)	20 (5–25)
Urinary protein(g/day)	2.33 (0.14–8.31)**	0.87 (0.15–5.53)	Cumulative consumption (pack × year)^b^	3 (1–38)	2.5 (1–38)
Hypertension [n (%)]	40 (52.3)**	13 (16.9)	Alcohol consumption		
Prednisone treatment [*n* (%)]	45 (58.4)*	30 (40.0)	Current/ex-drinkers [*n* (%)]	23 (29.9)*	11 (14.3)
Immunosuppression treatment^a^ [*n* (%)]	36 (46.8)	27 (35.1)	Drinking length (years)^c^	10 (3–30)*	2 (1–10)
RAS blockade treatment [*n* (%)]	62 (80.5)	67 (87.0)	Daily consumption (g/day)^c^	23 (2–250)*	3 (2–25)
			Physical exercise [*n* (%)]	19 (24.7)*	34 (44.2)

eGFR: estimated glomerular filtration rate, ml·min^−1^·(1.73 m^2^)^−1^; CKD: chronic kidney disease.

a Immunosuppression except for corticosteroids.

b Statistical objects for patients with cigarette smoking habit.

c Statistical objects for patients with alcohol consumption history.

* *p* < .05.

** *p* < .001 by the *t* test, Chi-square, or Mann–Whitney *U* test.

**Table 2.** Spearman’s correlation coefficients of clinical characteristics and lifestyles among cases and controls.

		Cigarette smoking status				Alcohol consumption status			Exercise
		Yes/No	Cigarettes per day	Years	Pack* Year	Yes/No	Alcohol g per day	Years	Yes/No
Gender	Cases	0.439**	0.428**	0.428**	0.428**	0.287**	0.308**	0.314**	0.162
	Controls	0.436**	0.434**	0.434**	0.434**	0.361**	0.357**	0.368**	0.163
Age	Cases	−0.107	−0.048	−0.119	−0.073	0.106	0.142	0.165	−0.239
	Controls	0.272*	0.270*	0.281*	0.278*	0.136	0.141	0.124	0.011
eGFR	Cases	0.085	0.088	0.048	0.039	−0.066	−0.071	−0.080	0.068
	Controls	−0.156	−0.161	−0.155	−0.145	0.184	0.186	0.191	−0.165
Proteinuria	Cases	0.004	0.009	−0.028	−0.018	−0.021	−0.005	0.026	0.008
	Controls	0.159	0.144	0.159	0.162	0.016	0.024	0.009	0.180

* *p* < .05.

** *p* < .001.

Associations of cigarette consumption (variables including current/ex-smoker, smoking years, daily consumption, and cumulative consumption), alcohol consumption (variables including current/ex-drinker, drinking years and daily consumption), exercise habits and the risk of ESRF were analyzed by univariate and multivariate logistic regression, respectively. Current/ex-smokers, current/ex-drinkers, having alcohol drinking history (years), and having regular exercise habits were identified significantly related to the risk of ESRF in univariate logistic analysis, but only having alcohol drinking history (/1 year, OR 1.32, *p* < .05) and physical exercise habits (OR 0.06, *p* < .05) maintained significant results when adjusted for confounding factors in multivariate analysis (Table 3). Then, we did a multivariate logistic analysis in the model contains the above two significant factors and confounding factors. The results showed that drinking years, physical exercise habits, gender, lower eGFR, and higher urinary protein at biopsy were strongly related to the risk of ESRF (Table 4). It was also worth noticing that compared with the OR value of gender in model 0, the figure changed by more than thirty percent when variables of alcohol consumption status (took drinking years for example) [OR (95% CI), 5.53 (1.70–18.00) versus 3.87 (1.12–13.33)] or exercise status [OR (95% CI), 5.53 (1.70–18.00) versus 11.00 (2.47–48.96)] entered the model, which also indicated that alcohol consumption and regular exercise contribute partly to the OR value of gender.

**Table 3.** Effects of lifestyle variables on progression to ESRF^a^.

	Univariate		Multivariate^b^		
Variables	OR (95%CI)		*p* Value	OR (95%CI)	*p* Value
Cigarette smoking					
Current/ex-smokers	2.89 (1.30–6.40)	.006			NS
Length (years)		NS			NS
Cigarettes per day/10		NS			NS
Pack × year		NS			NS
Alcohol consumption					
Current/ex-drinkers	2.56 (1.14-5.71)	.022			NS
Length (years)	1.23 (1.08–1.40)	.002		1.28 (1.01–1.61)	.039
Daily alcohol (10 g/d)		NS			NS
Physical exercise	0.43 (0.22–0.85)	.016		0.08 (0.02–0.37)	.001

a NS: not significant.

b Models adjusted for age, gender, eGFR, urinary protein, history of hypertension, and treatment of prednisone and immunosuppression.

**Table 4.** Univariate and multivariate logistic analysis of risk for developing ESRF^a^.

Parameter	Univariate		Multivariate	
	OR (95%CI)	*p* Value	OR (95%CI)	*p* Value
Gender (male)	2.85 (1.46–5.55)	.002	7.31 (1.54–34.76)	.012
eGFR	0.95 (0.94–0.97)	<.001	0.95 (0.92–0.98)	.001
Urinary protein	1.98 (1.46–2.67)	<.001	2.07 (1.20–3.58)	.009
Physical exercise	0.43 (0.22–0.85)	.016	0.06 (0.01–0.33)	.001
Drinking history length (years)	1.23 (1.08–1.4)	.002	1.32 (1.02–1.72)	.038
Prednisone treatment	2.35 (1.20–4.62)	.013		NS
Hypertension	5.43 (2.49–11.85)	<.001		NS
Age		NS		NS
Immunosuppression treatment		NS		NS

a NS: not significant.

Since there were significant associations among gender, smoking and alcohol consumption, we analyzed the correlation of smoking, alcohol drinking and risk of ESRF particularly in males (Table 5). There were no significant differences observed between males with or without the habit of smoking/alcohol drinking. Those who have both habits did not differ from those have-no in the OR value.

**Table 5.** Association of smoking and drinking with risk to ESRF in males.

Lifestyle factors	Observation case/control	Adjusted OR^a^ (95%CI)
Cigarette smoking		
Non-smoker	30/25	Ref
Smoker	25/11	1.12 (0.36–3.44)
Alcohol consumption		
Non-drinker	34/26	Ref
Drinker	21/10	1.80 (0.54–6.0)
Combined variable		
Non-smoker and drinker	26/20	Ref
Smoker only	8/6	0.39 (0.08–1.94)
Drinker only	4/5	0.39 (0.03–4.86)
Smoker and drinker	17/5	1.95 (0.48–7.94)

a Models were adjusted for age, eGFR and urinary protein at baseline.

To investigate the contributions of regular exercise did to the OR value of gender, we analyzed the associations between gender-exercise combined variables and risk of ESRF. As was shown in Table 6, compared with females having no exercise habit, females with such a habit have a lower risk of ESRF, but males without exercise habit were more likely to end up with ESRF (Model 1). The combination situation of male with no exercise habit shows a significantly worse renal outcome than the other three (Model 2).

**Table 6.** Gender-physical exercise combination on the risk to ESRF.

Gender	Physical exercise	Observation case/control	Model1 OR^a^ (95%CI)	Model 2 OR^b^ (95%CI)
Female	No	26/19	Ref	0.11 (0.03–0.37)**
	Yes	15/3	0.11 (0.01–0.94)*	0.01 (0.01–0.12)**
Male	No	17/39	9.16 (2.70–31.15)**	Ref
	Yes	19/16	1.62 (0.49–5.35)	0.18 (0.05–0.59)*

a Model 1 used female patients who had no exercise habit as the reference and was adjusted for age, eGFR and urinary protein at baseline.

b Model 2 used male patients who had no exercise habit as the reference and was adjusted for age, eGFR and urinary protein at baseline.

* *p* < .05.

** *p*< .01.

## Discussion

IgAN is recognized as one of the most common glomerulonephritis worldwide leading to renal failure. A few clinical and pathological features, such as baseline renal function, proteinuria and Oxford classification, were identified associated with the outcome of IgAN patients in previous studies. However, compared to these relatively unchangeable features, effective treatments and interventions may have more long-term significance. Recently, unhealthy lifestyles have drawn researchers’ attention. Cigarette smoking and alcohol consumption were reported significantly related to CKD prevalence and IgAN progression [4,6]. But there were few papers related to these kinds of issues on the Chinese population. On the other hand, variability in genetics, diet, or lifestyle in different geographical positions may also make some differences in renal outcomes [7,8]. It was still of value to investigate the potential impacts of those daily lifestyles on the progress of IgAN in the Chinese population. Three daily behaviors which we were most interested in, cigarette smoking, alcohol drinking, and taking regular exercise were discussed in this study.

For cigarette smoking, many studies had assessed the effect of smoking on renal function. The design and targeted samples extremely varied [3,9,10], which using surrogate markers of renal damage, renal function decline, urine albumin excretion rate, or renal failure. In general, smoking was identified as a risk factor for renal damage. In this study, we used an extreme clinical end point, ESRF, and gave the conclusion that smoking does not have a direct relationship with IgAN progressing to ESRF. But the possibility that smoking may have a mild but not an independent impact on renal function degradation cannot be excluded, and a large-scaled cohort study is still needed.

There were dissenting assessments about the influence of alcohol consumption on renal outcome in previous studies, and the variability in designs and outcome events were also considerable. A meta-analysis and some population-based cohorts research indicated that alcohol consumption was inversely associated with the risk of CKD [11–13]. However, a large-scale prospective cohort study illustrated that a small amount of alcohol consumption was linked to a higher risk to the decline of renal function [14]. In our study, the length of drinking years was identified independently associated with the increased risk of ESRF, but current or ex-alcohol drinkers and the average amount of alcohol consumed per day were not. Since previous studies mostly focused on the daily amount or frequency of drinking, this was the first time, as far as we learned, that drinking length was investigated and identified as a risk factor of ESRF, and indicated that alcohol abstinence may benefit the renal outcome.

Low self-reported physical function was reported associated with the development of CKD, because of the probable connection between physical inactivity and the greater prevalence of metabolic syndrome and elevated CRP [15]. Evidence showed that walking was beneficial to immune and inflammatory responses and had the potential to become an effective anti-inflammatory therapy in pre-dialysis CKD [16]. Data in our study supported the prognostic value of self-reported exercise on predicating good renal outcome in different aspects after adjusted for relevant confounders. First, patient having this healthy habit was less likely to develop ESRF than those not. Second, exercise men and non-exercise women did not differ in risk of ESRF. Third, the male-non-exercise population was the last group that can have a favorable outcome. Thus, we suggested that participating in physical activities regularly benefit against the progression of IgAN, especially for men.

Besides two widely acknowledged risk factors, decreased eGFR and high urinary protein at baseline, male gender was also identified as an independent risk factor for developing ESRF in this study. There were numbers of experimental and clinical researches having investigated the differences of the disease developing mechanism in males and females. In terms of renal diseases, sexual dimorphism was observed in the impact of angiotensin II (AngII) type 2 receptor (AT2R) on vascular responses to AngII in renal ischemia/reperfusion injury [17]. The ovarian hormones were identified as protectors for programed accelerated renal aging [18]. The effects of sex hormones seemed to partly contributed to the developmental programing of some diseases [19]. Besides potential physical mechanisms above, unhealthy lifestyle, diet, and psychological factors may also partly contribute to the risk of adverse outcomes of IgAN in males. In our study, the change of OR values of gender when alcohol consumption-related variables or physical exercise entered model 0 suggested that alcohol consumption and physical exercise may partly affect the influence of gender on renal outcome.

Our study had several limitations. First, the time span was long and may have resulted in heterogeneity and bias in the patient inclusion. Second, as the data of physical exercise prior to the time of biopsy were collected by the questionnaire on the phone, the recall bias was inevitable. Third, the number of the cases was relatively small and the rate of loss to follow up was not low. Fourth, as this was an observational study, the preventive effect of the improvement in lifestyles on slowing down the progress to ESRF has remained unclarified. Therefore, prospective cohort studies with larger sample size are urgently needed.

## Conclusion

Alcohol abstinence and taking physical exercise could be encouraged in IgAN patients, especially in males who were more likely to progress to ESRF than females.
